# ctDNA to Guide Adjuvant Therapy in Localized Colorectal Cancer (CRC)

**DOI:** 10.3390/cancers13122869

**Published:** 2021-06-08

**Authors:** Laura Masfarré, Joana Vidal, Concepción Fernández-Rodríguez, Clara Montagut

**Affiliations:** 1 Medical Oncology Department, Hospital del Mar, 08003 Barcelona, Spain; lmasfarre@psmar.cat (L.M.); jvidal@psmar.cat (J.V.); 2 Cancer Research Program, FIMIM, Hospital del Mar, 08003 Barcelona, Spain; 3 Pathology Department, Hospital del Mar, 08003 Barcelona, Spain; mconcepcionfernandezrodriguez@psmar.cat

**Keywords:** liquid biopsy, circulating tumor DNA, minimal residual disease, colorectal cancer, next-generation sequencing, cancer detection

## Abstract

**Simple Summary:**

The assessment of risk of recurrence following surgery in patients with localized colorectal cancer (CRC) is crucial to indicate systemic adjuvant therapy. The presence of circulating tumor (ct)DNA in the plasma of patients after treatment with curative intent has recently been defined as minimal residual disease (MRD). Detection of MRD is a powerful prognostic biomarker which reflects the presence of micrometastasis and can potentially guide the need of systemic treatment before becoming clinically evident. The aim of this review was to highlight and explore the current situation of MRD detection in CRC cancer and its potential impact in routine clinical practice.

**Abstract:**

Currently, the standard treatment for patients with localized colorectal cancer (CRC) includes surgical resection followed by adjuvant chemotherapy based on clinicopathological features. Recurrence risk stratification in those patients is of utmost importance to guide clinicians to avoid both under- and overtreatment. Recently, the concept of minimal residual disease (MRD) has emerged as the detection of circulating tumor DNA (ctDNA) carrying tumor-specific genomic or epigenomic alterations in the bloodstream of patients after surgery. Emerging studies described how the detection of MRD is a powerful prognostic biomarker to identify patients at higher risk of recurrence and who will potentially benefit the most from a systemic adjuvant treatment. Based on that unprecedented finding, several clinical trials involving stage II and III CRC patients are ongoing evaluating the impact of ctDNA guided treatment by escalating or deescalating adjuvant chemotherapy based on ctDNA MRD detection. This review provides a critical overview of current perspectives of liquid biopsy in early-stage CRC including technical, biological, and clinical key points, as well as ongoing ctDNA-based clinical trials that ultimately aim to improve clinical outcomes of patients with CRC.

## 1. Introduction

### Current Perspectives of Liquid Biopsy in Colorectal Cancer (CRC)

Colorectal cancer (CRC) is the third most frequent cancer in men and the second in women. The prognosis depends not only on the stage at diagnosis, but also the surgical alternatives and the systemic treatment received. Due to the implementation of screening programs, the introduction of novel systemic therapies, and the advanced surgical procedures, the oncological outcomes have dramatically improved in the last years. However, CRC is still the second leading cause of cancer-related mortality worldwide [1].

In patients with localized CC, the assessment of risk of recurrence following surgery is a crucial point to indicate systemic adjuvant therapy. This assessment is based on tumor TNM staging and other clinicopathological characteristics including CEA status or lymphovascular and perineural invasion [2]. Currently, standard fluoropyrimidine-based therapies estimate an increase in overall survival of approximately 5% in stage II patients and 20% in stage III patients when adding oxaliplatin [3,4]. However, at least 50% of patients with stages I, II, and III treated with curative-intent surgery receives unnecessary adjuvant chemotherapy [5,6]. On the other hand, there is a small proportion of patients with stages I and II who do not receive adjuvant treatment and recur [5,6].

Treatment paradigm is slightly different in locally advanced rectal cancer (LARC). Patients with clinical stage T3/4 or node-positive tumors are treated with neoadjuvant chemo-radiotherapy (CRT) or total neoadjuvant treatment (TNT) [7,8] followed by surgery. For those patients who received neoadjuvant CRT, the role of adjuvant chemotherapy is controversial, and FOLFOX only demonstrated benefit in stage III pathological tumors [9,10].

Moreover, it is essential to consider that these treatments are not exempt from adverse effects, such as digestive toxicity or palmoplantar erythrodysesthesia which can be fatal in a small subset of patients carrying a DPYD polymorphism [11], or peripheral neuropathy caused by oxaliplatin that limits day-to-day activity and is permanent in at least 10% of patients [12,13].

Thus, determining the presence or absence of minimal residual disease (MRD) is of utmost importance to guide clinicians to avoid both under- and overtreatment in the adjuvant setting. Circulating cell-free DNA (cfDNA) is a highly fragmented DNA mainly derived from apoptotic cells, predominantly apoptotic leukocytes, found in the blood. cfDNA concentrations in healthy individuals range between 1 and 10 ng mL^−^^1^ in plasma [14,15]. Circulating tumor DNA (ctDNA) is a fraction of cfDNA characterized by the presence of tumor-specific genomic alterations with a half-life of a few hours allowing for a real-time, non-invasive characterization of the tumor molecular profile.

ctDNA allows for a better and less aggressive characterization of the spatial tumor molecular heterogeneity and its temporal evolution compared to the traditional use of tissue tumor biopsy. ctDNA, although generally obtained from peripheral blood, can also be isolated from other fluids such as urine, saliva, or cerebrospinal fluid [16,17,18,19,20]. In the metastatic setting, a high concordance between detection of mutations in tissue tumor compared to ctDNA has been reported [21,22,23]. However, ctDNA represents between 0.005% and 11.7% of the whole cfDNA shed in the bloodstream [24] depending on the tumor size, tumor growth rate, and cell turnover [25]. During the early stages of cancer, the total amount of ctDNA might be <1% of the total cfDNA concentration according to some studies [24,26,27]. These extremely low concentrations of ctDNA have been one of the main challenges of liquid biopsies in early-stage disease.

Recently, there have been several advances in the development of new technologies for the detection of ctDNA. Current techniques allow for the detection of genomic molecular alterations (including point mutations, short insertions and deletions, copy number alterations, and fusions), as well as epigenomic changes (i.e., methylation) and cfDNA fragmentation pattern identification. The increased sensitivity in the detection of these biomarkers is crucial for an accurate detection of MRD in CRC cancer patients.

Several ongoing clinical trials are evaluating the use of ctDNA to guide adjuvant therapeutic strategies in CRC patients after curative-intent surgery, and will ultimately shed light onto whether ctDNA is a useful tool to improve outcomes of localized CRC patients. This review highlights and explores the current situation on this topic and its potential application in routine clinical practice.

## 2. Technical Approaches in Detecting MRD

The molecular landscape of colorectal cancer has been well characterized in the past decades, including chromosomal aberrations such as copy numbers alterations (CNAs), inversions, translocations, insertions, and deletions, as well as single nucleotide point mutations [28]. Epigenomics, referring to covalent modifications of DNA that result in a change in its function or in the regulation of the affected genes without altering the primary sequence, has also been well described in colorectal cancer. These molecular alterations are highly specific to cancer, and, thus, their detection in an individual’s blood potentially indicates the presence of cancer. The extremely low concentration of ctDNA (approximately 0.01% of total cfDNA) especially in early stages of the disease makes its detection challenging [29,30,31].

Currently, two main strategies are used to study tumor genomic material in ctDNA for MRD detection after curative-intent surgery. On the one hand, techniques based on the detection of one or several mutations previously found in the primary tissue tumor. These techniques include sensitive qPCR based methods, such as ARMS or COLD-PCR as well as digital PCR-based methods, such as ddPCR or BEAMing among others [32]. This strategy has the main disadvantage of needing detailed information of the primary tumor (mutation needs to be known ahead of time) and that only a limited number of known mutations can be tracked in the blood (although multiplexing assay is possible). However, it is a very sensitive (0.01%) and specific technique in addition to being rapid and cost-effective [33,34,35].

**Figure 1:**
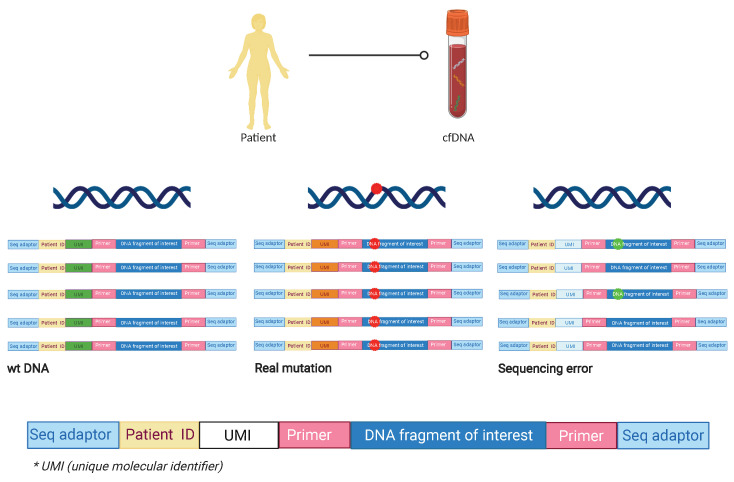
Preparation of sequencing libraries using ID barcodes and unique molecular identifiers (UMI). After the area of interest is selected, the ID barcodes specific to each patient are added to enable to analyze multiple patients in the same assay. After that, UMIs are added to each molecule during library preparation so that sequencing reads originating from the same starting molecule of each patient included in the library can be identified. This approach increases the assay sensitivity in terms of detecting sequencing errors. In the right sequence corresponding to the blue UMI, the alteration is objectified in only 2 of the amplifications, so it is assumed a sequencing error. In the middle sequence corresponding to the orange UMI, the mutation is found in all the amplifications so it is assumed a real mutation. In the left sequence, there is no mutation founded in any of the reads, so it is a wt ctDNA molecule. cfDNA: cell free DNA; wt: wild type; ID: identification; * UMI: unique molecular identifier.

The second strategy, the so-called deep next-generation sequencing (NGS), allows for the detection of multiple genetic alterations in one sample. NGS conducts a non-directed scan by analyzing the entire genome to detect CNAs or point mutations through whole genome sequencing (WGS) or exome sequencing (WES) [36]. The technique can be PCR or capture-based which allows for the reading of short fragments (around 150 bp). NGS has a high false discovery rate that requires pre-sequencing barcoding and post-sequencing bioinformatics for error suppression. In order to obtain a high sensitivity (0.1%), it is necessary to perform DNA barcodes or UMIs (unique molecular identifiers), initial DNA molecules with 10–12 random bases, such that the barcode is amplified and sequenced together with the DNA. Thus, if we find an alteration, and it is present in all the sequences of the same patient with the same UMI, it will be considered a real alteration, whereas if it is only found in one of the sequences it will be considered as a sequencing failure (Figure 1) [37,38,39].

NGS has several advantages, including the ability to detect molecular alterations that emerge during treatment, and the fact that it is not necessary to have molecular information on the primary tumor. Potential limitations include the requirement for higher amounts of cfDNA [40] (20–50 ng of cfDNA) to decrease the presence of false negative results. Another challenge is the process known as clonal hematopoiesis (CH). Healthy individuals, with age, acquire new mutations in hematopoietic cells [41] and, when lysed, can release cfDNA carrying somatic mutations. This can be incorrectly interpreted as the presence of ctDNA from tumor cells. Razavi et al. [42] sought to develop a protocol for the distinction between mutations of tumor origin and those of hematological origin. They analyzed 124 patients with different types of metastatic tumor and in all cases parallelly sequenced a primary tumor tissue sample, cfDNA, and white blood cells. The authors found that half of the mutations identified in cfDNA of cancer patients were originated in the hematological compartment as a result of clonal hematopoiesis and were therefore not derived from the primary tumor. This study shows the need to sequence the leukocyte fraction in order to discriminate the origin of the mutation detected in cfDNA. All of this is of utmost relevance in the detection of MRD and in cancer screening in which a false positive result may have important consequences.

Currently, new strategies are being studied to increase the sensitivity of diagnostic techniques. These include the analysis of methylation tumor profile via nucleosomal positioning or epigenomic alterations at transcription factor binding sites [43,44]. Methylation patterns that inhibit gene expression are related to the tissue to which they originally belong and therefore reflect the origin of circulating DNA. When a gene is methylated, it does so in various positions of the promoter, thus increasing the opportunity to detect these regions and thus increasing the sensitivity of the technique. This approach has been recently explored by detecting ctDNA using two methylation markers (WIFI and NPY) by ddPCR in CRC patients [45,46].

New NGS strategies are arising by combining different methods to improve ctDNA detection. Such is the case of LUNAR-1 technology (Guardant Reveal^TM^ Guardant Health), which integrates assessment of somatic alterations with an epigenomic cancer signature without a priori knowledge of tumor mutation. Sequencing data files are analyzed using a proprietary bioinformatics pipeline software to exclude common sources of interference such as CH of indeterminate potential [47]. Novel NGS assays are increasing the sensitivity to detect ctDNA in MRD. However, the need of bioinformatics analysis to exclude eventual false negative results increases the complexity and subsequently its costs and turnaround times.

**Figure 2:**
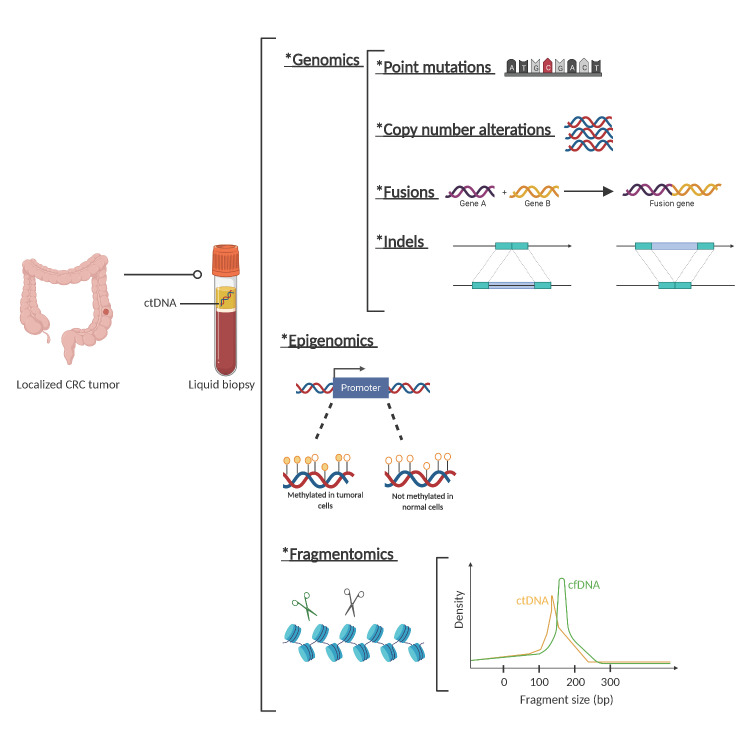
Circulating tumor (ct)DNA features. Description of genetic, epigenetic, and fragmentomic alterations that can be found in plasma cell free DNA analysis. *: The asterisks are placed as a way of listing the different techniques.

Regarding fragmentation, it is well described that the fragmentation cut-off points of circulating DNA are not random and are different for ctDNA compared to cfDNA from normal cells. While the mechanisms of this different fragmentation pattern are yet unknown, they help to characterize whether cfDNA is of tumoral origin or not (Figure 2).

Emerging data suggest that elucidating nucleosome positioning opens promising new perspectives to identify the tissue source of origin of cancer from cfDNA, with an important clinical value to classify cancers and, to a further extent, to characterize, for example, cancers of unknown origin [48].

Single biomarkers in liquid biopsy often do not accurately predict disease status due to heterogeneity between individuals. To address this challenge, investigators are combining multiplexed measurements of different biomarkers that together define robust signatures for specific disease states. Machine learning is a useful tool to automatically discover and detect these signatures, especially as new technologies output increasing quantities of molecular data. Machine learning approaches have been proposed to classify the cell of origin based on somatic mutation profiles in the genome of solid tissue biopsies. Therefore, it is crucial to investigate the applicability of sparse somatic mutation profiles in the identification of ‘cell of origin’ and explore potential improvements of the data analysis and prediction models to overcome sparsity [49].

With only few years, liquid biopsy has rapidly evolved from detecting point mutations in known given genes, to large genome sequencing and the detection of methylations and fragmentomics. This dramatic change has allowed us to improve ctDNA detection sensitivity and even avoid tissue information and carry out molecular studies only on liquid biopsy. However, this entails an increase in the complexity of the analysis and therefore in cost and time to results. Despite these technical improvements, liquid biopsies still have some biological limitations. In the metastatic CRC setting, the discordance in the finding of mutations between tissue and plasma has been shown to be related to the location of the metastasis (pulmonary or peritoneal) and the histology of the tumor (predominantly mucinous) [22,50,51].

## 3. ctDNA Detection in Localized CRC

The presence of ctDNA after curative-intent surgery in patients with localized disease has been very consistently associated with a high risk of recurrence in different tumor types [52,53,54,55]. Several observational studies have shown that ctDNA detects MRD and is associated with recurrence in patients with localized CRC that have undergone surgery of the primary tumor. In these studies, ctDNA detection typically precedes the appearance of both clinical and radiological recurrence by an average of 3–5 months [56,57,58,59]. In addition, ctDNA has been shown to also be a useful tool for patients with metastatic CRC receiving multi-therapy with curative intention [24]. Interestingly, subjects with detectable ctDNA after surgery generally relapsed within 1 year, while patients who completely negativized ctDNA after surgery have a much lower probability of recurrence and eventual relapse tends to occur later in time.

### 3.1. Stage II Colon Cancer

Up to 25% of newly diagnosed CRC cases are stage II. Data from clinical trials show that up to 80% of patients who receive surgery with curative intent do not recur [60] and are therefore cured. While TNM staging remains the most relevant criteria for risk assessment after surgery, in stage II CC other clinicopathological parameters need consideration to decide the need for adjuvant chemotherapy [2]. Major prognostic factors include the pT4 stage and perforation and lymph nodes sampling < 12. Minor prognostic parameters for stage II risk assessment include high histological grade, lymphatic, vascular, or perineural invasion, tumor presentation with obstruction or high preoperative CEA levels [2,61,62]. MMR/MSI status is also a validated prognostic marker. MSI/MMR status defines, in stage II CC, a subgroup of patients with a better prognosis and less expected benefit from chemotherapy [63,64,65]. Adjuvant systemic therapy is recommended in stage II CC with at least one risk factor. However, a survival benefit of adjuvant chemotherapy in high-risk stage II CC has not been conclusively demonstrated [66,67]. Identification of an accurate prognostic biomarker would help for a better selection of patients that benefit from adjuvant systemic therapy, and spare chemotherapy in cured patients.

With the aim to use ctDNA as a prognostic biomarker, Tie et al. [56] studied the impact of post-operative ctDNA detection in stage II colon cancer patients.

A total of 250 patients with stage II CC were included, in which a blood sample with ctDNA and CEA analysis at 4–10 weeks post-surgery (post-IQ) and subsequently every 3 months for 2 years was performed. Patients were given chemotherapy at the investigator’s criteria and a clinical follow-up was performed every 3 months including CT imaging tests every 6 months for 2 years. During the follow-up, 34 patients out of 230 (14.8%) had radiological recurrence, including 27 of 178 (15%) patients not treated with chemotherapy and 7 of 52 (13%) patients treated with adjuvant chemotherapy.

Seventy-eight percent (11 out of 14 patients) of patients who did not receive adjuvant treatment and had post-surgery positive ctDNA had radiological recurrence, whereas only 16 out of 164 (9.8%) ctDNA negative patients recurred. CEA was elevated in 7.4% of recurrent patients (2 of 27 cases) and none of the patients with positive ctDNA had an increase in CEA after surgery. Recurrence-free survival (RFS) was estimated to be 0% at 3 years in patients with post-surgery positive ctDNA, compared to 90% in patients with post-surgery negative ctDNA (HR 18; 95% CI 7.9 to 40; *p* = 2.6 × 10^−12^). Post-surgery ctDNA was found to be the most significant prognostic independent factor associated with RFS. Finally, it was observed that the time between the detection of ctDNA in peripheral blood and radiological recurrence was on average 167 days, much longer than in the case of CEA which was 61 days.

The use of ctDNA was shown to be superior to the currently used clinicopathological risk parameters to decide on adjuvant treatment. On the other hand, the probability of recurrence was very low for patients with ctDNA negative, defining a subset of patients that could avoid adjuvant chemotherapy and potential associated toxicities.

Combining the use of ctDNA with imaging tests in the follow-up of these patients may help in the early detection of disease recurrence and potentially ultimately lead to better results in terms of survival [68,69].

### 3.2. Stage III Colon Cancer

Randomized phase III clinical trials have shown that the use of adjuvant chemotherapy with oxaliplatin plus a fluoropyrimidine (FOLFOX or XELOX regimens) improves the overall survival of patients with stage III CC [70]. Most of these patients receive chemotherapy, although up to 50% of them are cured by surgery, and chemotherapy could be potentially be spared [71,72]. Among patients receiving chemotherapy, approximately 30% will have recurrence of the disease and are potential candidates for other systemic treatments [73,74].

Retrospective subgroup analysis of large randomized clinical trials supported a different duration of adjuvant FOLFOX/XELOX treatment depending on pathological risk factors. Thus, in patients considered to be at low risk (≤T3 and N1) the duration of the adjuvant treatment of 3 months is considered appropriate, whereas in the case of patients with high-risk tumors (T4 and/or N2), the duration of treatment is recommended to be extended to 6 months [75].

Reinert and colleagues [76] carried out a prospective and observational study analyzing ctDNA as a biomarker in patients with stage I to III CRC (mostly stage III) with the aim of demonstrating that the presence of post-surgery ctDNA is related to a high probability of recurrence. The study enrolled 130 patients and plasma samples were collected before surgery, after 30 days, and then every 3 months up to 3 years. The recurrence rate was 70% (7 out of 10 patients) in patients with positive ctDNA after surgery, compared to 11.9% (10 patients out of 84) for patients with no ctDNA detection. After surgery, patients with ctDNA were 7 times more likely to relapse than ctDNA-negative patients (HR 7.2; 95% CI 2.7–19.0; *p* < 0.001). After a multivariate analysis in which known clinical-pathological risk factors, such as staging and lymphovascular invasion, were included, the detection of ctDNA was the only statistically significant prognostic factor associated with RFS.

In line with the aforementioned, the Australian team led by Tie [77] has published the results of a multicentric clinical trial in which 100 patients with a diagnosis of CRC stage III with the provision of administering adjuvant treatment with chemotherapy for 6 months were consecutively recruited. Samples were collected from the primary tumor and later peripheral blood samples for ctDNA determination 4–10 weeks post-surgery and later after completion of adjuvant treatment. The aim of the study was to find whether the determination of ctDNA post-surgery and after completion of adjuvant chemotherapy treatment may give information on minimal residual disease, the efficacy of adjuvant treatment, and recurrence in patients with stage III CRC.

ctDNA was detected in 20 out of 96 (21%) post-surgery patients and was associated with a decrease in RFS (HR 3.8; 95% CI 2.4–21.0; *p* < 0.001). On the other hand, ctDNA was detected in 10 out of 66 patients (15%) after finishing treatment with adjuvant chemotherapy. Detection of ctDNA after completion of adjuvant treatment was significantly associated with recurrence free interval (RFI). RFI at 3 years was 30% in patients with detectable ctDNA and 77% in patients with negative ctDNA after chemotherapy (HR 6.8; 95% CI, 11.0–157.0; *p* < 0.001). ctDNA status was the prognostic factor most strongly associated with RFI.

Recently, the same group published a pooled analysis of three cohort studies including 485 stage II–III CRC patients with long term follow-up of 5-years after-surgery ctDNA collection [78]. The authors describe the association of post-surgery ctDNA detection and higher risk of recurrence (38.6% vs. 85.5%; *p* < 0.001) and poorer OS (64.6% vs. 89.4%; *p* < 0.001). Furthermore, post-surgery ctDNA status was more accurate in predicting recurrence than individual clinical-pathological risk features such as tumor differentiation, T stage, N stage, lymphovascular invasion, and post-surgery CEA.

All published studies are consistent regarding the clinical impact of ctDNA to detect MRD in stage III CC, not only in identifying patients at high risk of recurrence, but also guiding clinical trials to explore new adjuvant approaches for patients with detectable ctDNA after surgical resection.

Table 1 summarizes the main published studies assessing ctDNA prognostic role in localized CC.

### 3.3. Locally Advanced Rectal Cancer (LARC)

As previously mentioned, CRC is a health problem of global importance. Among patients diagnosed with CRC, 30% have a rectal location [1]. Patients with high-risk LARC (defined by either clinical stage T3/4 or node-positive disease) are usually treated by neoadjuvant CRT or TNT followed by total mesorectal excision (TME) surgery (a mutilating procedure with significant alteration in the quality of life of patients). Moreover, the main site of recurrence in LARC patients is not local but distant metastasis which causes greater morbidity and mortality [10].

Currently, the main prognostic marker in LARC patients is pathological complete response (pCR) as assessed in the rectal surgical specimen after neoadjuvant treatment [81,82].

The need to avoid such mutilating surgery with the associated morbidity has led to an increased interest in the search for prognostic factors to select candidate patients for organ preservation [83]. Currently, watch-and-wait strategies are a possibility in patients with clinical and radiological complete response after TNT. In this setting, the use of ctDNA may potentially help in better identifying patients that are cured after total neoadjuvant therapy and that are potential candidates for a watch-and-wait strategy.

The Australian team led by Tie [84] published a prospective multicenter study that included 159 patients with LARC treated with neoadjuvant CRT followed by TME. Samples were collected at baseline, after completing CRT and 4–10 weeks after surgery. ctDNA was detectable in 77%, 8.3%, and 12% of pretreatment, post-CRT, and post-surgery plasma samples, respectively. Patients with detectable ctDNA after CRT or after surgery had a significantly worse RFS irrespective of receiving or not adjuvant CT (HR 6.6; 95% CI 2.6–17; *p* < 0.001 and HR 13.0; 95% CI 5.5–31; *p* < 0.001) respectively. No association was found between post-CRT ctDNA status and pCR.

In the same year, Khakoo and colleagues [85] investigated the use of ctDNA combined with MRI as an early indicator of response in 47 patients with LARC treated with neoadjuvant CRT followed by TME. Blood samples for ctDNA detection were obtained before treatment, during CRT, after completion of CRT and after-surgery. Metastatic disease was observed in 70% of patients with positive ctDNA after completing CRT and in 100% of patients with post-surgery positive ctDNA. Furthermore, metastasis-free survival (MFS) was significantly lower in patients with persistent ctDNA after completing CRT compared with patients with undetectable or non-persistent ctDNA (HR 7.1 95% CI 2.4–21.5, *p* < 0.001). However, no correlation was found between post-CRT ctDNA and pCR rate.

Accordingly, Zhou et al. [86] recently published a prospective multicentric study with the aim of analyzing the value of ctDNA in predicting response to neoadjuvant CRT. ctDNA from 104 patients was extracted and analyzed by NGS at four time points: baseline, during neoadjuvant CRT treatment, before surgery, and after surgery. With a median follow-up of 18.8 months, 12.5% of patients developed distant metastases. Positive ctDNA at all four time points was associated with decreased MFS. Moreover, variant allele frequency (VAF) of baseline ctDNA mutations was found to be a significant independent predictor of MFS (HR, 1.27; *p* < 0.001).

Murahashi et al. [87] studied plasma at baseline and after CRT from 85 patients and found that variations in ctDNA was an independent predictor of complete response to preoperative therapy (*p* = 0.0276).

Similarly, Pazdirek et al. [88] investigated changes in ctDNA levels of 36 patients with LARC undergoing neoadjuvant CRT and their relationship to treatment response. Positive ctDNA at baseline was associated with lower DFS and OS at 1.47 and 1.41 years respectively (*p* = 0.015 and *p* = 0.010, respectively).

Table 2 summarizes the main published studies assessing ctDNA prognostic role in LARC.

In contrast to the previously described studies, Vidal et al. [89] have recently published a study in which the concept of MMD (minimal metastatic disease) was introduced. This term refers to the detection of ctDNA following TNT and before surgery in patients that are likely to recur at distant sites. Plasma samples were collected at baseline and after TNT within 48 h before surgery (pre-surgery). Patients with pre-surgery positive ctDNA had an increased risk of recurrence compared to patients with negative ctDNA (HR 4.029; 95% CI, 1.004–16.16 *p* = 0.033) and a marked reduced survival (HR 23; 95% CI, 2.4–212 *p* < 0.0001). Interestingly, ctDNA was able to predict distant recurrence involving the liver more than metastasis to the peritoneum only or the lung only. In line with previous works in LARC, no correlation was found between pre-surgery ctDNA and pCR.

Globally, in LARC there are three timepoints where ctDNA has been mostly interrogated: baseline, after neoadjuvant treatment, and post-surgery. Data are inconclusive regarding the utility of baseline ctDNA. The prognostic role of post-surgery ctDNA is consistent with previous studies in CC and confirms the value of ctDNA to detect MRD. This finding is important because the clinical benefit of adjuvant therapy in patients with rectal cancer receiving neoadjuvant CRT has not yet been established, and adjuvant clinical trials based on post-operative ctDNA may help to answer this crucial question. Finally, pre-surgery ctDNA analysis after neoadjuvant treatment (either CRT or TNT) detects minimal metastatic disease (MMD), systemic recurrence and death. Prospective clinical trials are needed to validate these finding by assessing systemic treatment intensification in pre-surgery ctDNA positive patients. Interestingly, no association between ctDNA and pCR rates was found, potentially limiting the use of ctDNA alone to select for an organ-preserving approach.

## 4. ctDNA as a Post-Treatment Surveillance Strategy

Routine follow-up up of localized CRC includes clinical examination, serial CT-scan, blood tests, and CEA. However, when recurrence is detected, it is usually too late to give a curative treatment. ctDNA may potentially monitor adjuvant chemotherapy efficacy or detect resistance after treatment before clinical or radiological evidence of relapse.

In the study by Tie et al. [56] in patients with CRC stage II, assessment of ctDNA at the end of adjuvant therapy showed that a positive result was predictive of disease recurrence (HR 11, 95% CI 1.8–68). Similarly, in the study by Reinert et al. [76], ctDNA levels at the end of CT were correlated with recurrence in patients with CRC stages I–III. In this study, ctDNA after adjuvant chemotherapy was associated with disease recurrence with a sensitivity of 88% and specificity of 98%, whereas CEA had a sensitivity of 69% and specificity of 64%. ctDNA was analyzed every 3 months during follow-up. During surveillance, ctDNA-positive patients were 43 times more likely to experience disease recurrence than ctDNA-negative patients (HR 43.5; 95% CI 9.8–193.5; *p* < 0.001). The time from detection of ctDNA to detection of recurrence by imaging test was 8.7 months, as opposed to CEA, which rose at the same time as imaging recurrence was detected. At the time of radiological recurrence, an increase in the VAF of ctDNA from all patients, up to 300-fold, was also observed. Tarazona et al. [79] also monitored ctDNA after adjuvant treatment every 4 months for up to 5 years. ctDNA positivity after completion of CT was associated with poorer DFS (HR 10.02; CI 9.202–307.3; *p* < 0.0001) and detection of ctDNA preceded radiological relapse by a median of 11.5 months.

Interestingly, Reinert et al. [76] investigated whether ctDNA detects the presence of driver mutations to potentially guide administration of targeted personalized treatment in patients with detectable ctDNA after adjuvant therapy. Driver mutations were detected in 81.8% of the post-adjuvant ctDNA positive patients, concluding that ctDNA may serve as a biomarker to detect actionable mutations.

The detection of ctDNA, after the completion of adjuvant treatment, identifies patients that are refractory to standard adjuvant therapy and have poorer prognosis. This reflects the presence of molecular metastatic disease that is not yet identified in imaging tests. Compared to other currently used tests, such as CEA or imaging tests (i.e., computed tomography (CT)), ctDNA monitoring has shown an earlier detection of recurrence (median 9 months) [90,91]. Future studies should aim to determine whether a switch in chemotherapy regimen or administration of targeted therapy at the time of ctDNA detection ultimately impacts the clinical outcome of patients.

## 5. ctDNA Based Clinical Trials

**Figure 3:**
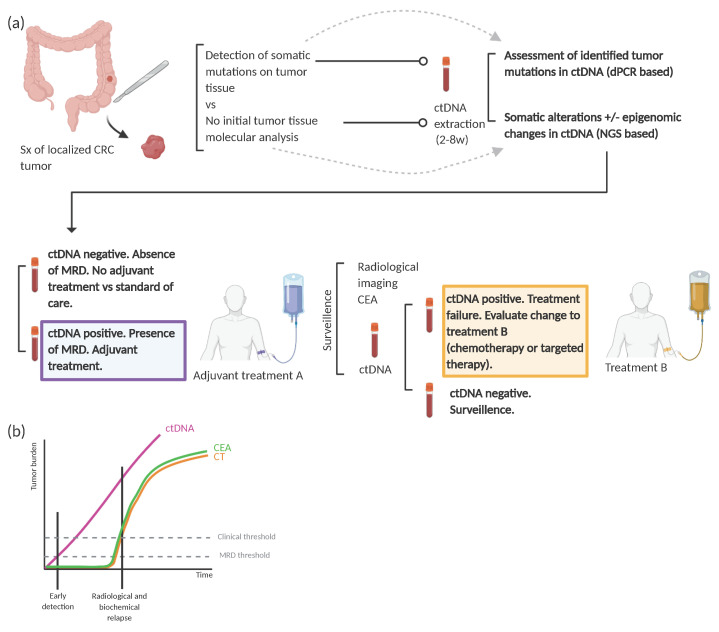
ctDNA detection of Minimal residual disease in colorectal cancer. (**a**) Design proposal for adjuvant clinical trials based on ctDNA analysis to detect MRD after surgery in localized colorectal cancer. (**b**) Graphical representation of early detection of MRD thanks to ctDNA compared to commonly surveillance used methods (CT scan, CEA). Sx: surgery; CRC: colorectal cancer; dPCR: digital PCR; NGS: next-generation sequencing; MRD: minimal residual disease; CT: computerized tomography; CEA: carcinoembryonic antigen.

Clinical implementation of liquid biopsies in localized CRC need prospective randomized clinical trials that show a meaningful impact in clinical outcome in the use of ctDNA to guide adjuvant treatment. Different study designs need to evaluate different clinical uses of ctDNA, including [92]: (a) ctDNA as a tool to detect MRD after curative-intent surgery and guide personalization of adjuvant therapy (intensification versus watch and wait strategy), (b) serial longitudinal ctDNA extractions to monitor and detect early recurrence of the disease (molecular relapse (MR)), (c) ctDNA to guide systemic therapy (i.e., chemotherapy, targeted therapy) in ctDNA positive patients after completion of adjuvant standard therapy (molecular metastatic disease) (Figure 3). Of note, the best time frame for the extraction of ctDNA samples (both in the search for MRD and for MR) has not been established yet.

**Figure 4:**
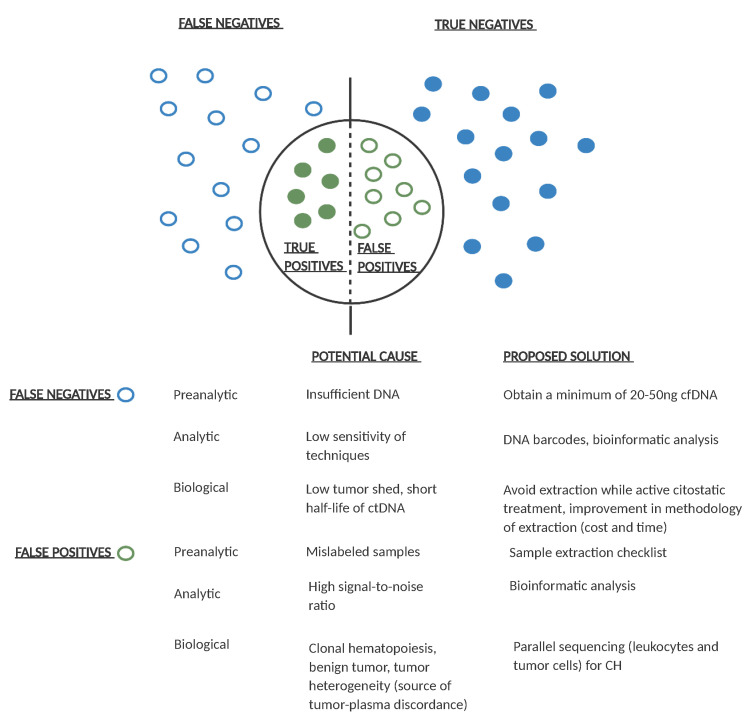
Potential causes of false positive and false negative results in ctDNA analysis and proposed solutions. Empty dots represent false positive/negative results and filled dots represent true positive/negative ctDNA results. For each pre-analytical, analytical, or biological potential false positive/negative result, a possible solution is proposed. Ng: nanograms; cfDNA: cell free DNA; CH: clonal hematopoiesis.

There are several challenges in the design of ctDNA-based clinical trials in adjuvant CRC. A major challenge is the requirement of a high number of patients in order to observe a significant clinical effect. A second challenge is the lack of standardization of ctDNA detection techniques [93], with different sensitivity and specificity. Specificity can be affected by multiple factors. A short follow-up can be related to false negative results. Other factors include clonal hematopoiesis or the presence of another primary tumor [94] which can lead to false positive results. Sensitivity is also highly variable among studies [24,80], and may be due to multiple factors including study design, temporal differences in the extraction of the first post-treatment sample, and differences in patient follow-up. A determining factor is the low amount of ctDNA in patients with localized disease [56]. Sensitivity has been shown to increase with serial sample extraction compared to one time-point extraction [55,80,95]. Figure 4 shows the main causes of false positive and false negative results and some proposed solutions.

Retrospective analysis of the impact of ctDNA in large randomized clinical trials would help define the clinical utility of ctDNA in the adjuvant setting. In this regard, a retrospective analysis of the French IDEA study showed that ctDNA-positive patients benefited from 6 months of chemotherapy compare to 3 months [46]. Prospective trials for the detection of MRD and its use to guide therapeutic decisions are ongoing. Currently, most of ongoing trials are designed to evaluate ctDNA as a tool to detect MRD after surgery and guide adjuvant therapy, while some of them are also addressing the question of chemo-resistant clones or persistent MRD following adjuvant chemotherapy (Table 3).

To sum up, in order to incorporate the use of ctDNA into routine clinical practice, clinical trials with robust and standardized methodologies are needed. The detection of MRD and MR by ctDNA needs to be proven of clinical utility to increase survival and/or reduce toxicities without affecting their long-term survival.

## 6. Conclusions

ctDNA-based liquid biopsy is changing the paradigm in the diagnosis and monitoring of CRC patients. Initially developed in the metastatic setting, the potential applications in MRD detection can change the treatment of patients with localized CRC. Studies carried out in recent years have validated the role of liquid biopsy as a powerful tool to detect MRD and a prognostic biomarker in patients with early-stage CRC. Thus, the detection of ctDNA after surgery correlates with a high risk of recurrence. Of note, most studies so far have been designed based on plasma testing of point mutations previously found in tumor tissue. The complexity of this approach may limit the implementation of ctDNA for MRD in clinical practice. With recent improvements in ctDNA technology, not only genomic but also epigenetic changes or fragmentomic can be used to detect ctDNA. This increases sensitivity and specificity in ctDNA detection, which is of utmost importance in the post-surgery clinical scenario where minimum amount of ctDNA is circulating in the bloodstream.

Results from ongoing ctDNA-guided prospective clinical trials in the adjuvant setting are eagerly awaited. Multiple efforts by cooperative research groups are being made to demonstrate the clinical impact of treatment intervention based on post-surgery ctDNA risk-stratification which will be essential to change the way we treat stage II and III CRC, as well as LARC.

## Figures and Tables

**Table 1:** ctDNA as prognostic marker in early-stage colorectal cancer.

Study	Sample Size	Study Population	Timepoint of ctDNA Collection	ctDNA Detection Assay	Post-op ctDNA Detection Rate	RFS Post-op ctDNA+ vs. ctDNA−
Tie et al. [56]	230	Stage II CC	Weeks 4–10 post-op	Safe-SeqS (1 variant; 15 genes)	8.7%	18 (95% CI 7.9–40) *p* < 0.001
Taieb et al. [46]	805	Stage III CC	NA	ddPCR (2 methylated markers)	13.5%	1.85 (95% CI 1.31–2.61) *p* < 0.001
Wang et al. [59]	58	Stage I–III CRC	Week 4 post-op	Safe-SeqS (1 variant; 15 genes)	22.4%	Recurrence-free at 49 months: 33% vs. 100% (non-compared)
Reinert et al. [76]	130	Stage I–III CRC	Week 4 post-op	Multiplex PCR based NGS assay (Signatera^TM^)	10.6%	7.2 (95% CI 2.7–19.0) *p* < 0.001
Tie et al. [77]	96	Stage III CC (all chemo)	Weeks 4–10 post-op	Safe SeqS (1 variant; 15 genes)	21%	3.8 (95% CI 2.4–21.0) *p* < 0.001
Tie et al. [78]	485	Stage II–III CRC and LARC	Weeks 4–10 post-op	Safe-SeqS (1 variant; 15 genes)	12%	Recurrence-free at 5 years: 38.6% vs. 85.5% *p* < 0.001
Tarazona et al. [79]	69	Stage I–III CC	Weeks 6–8 post-op	ddPCR (2 variants; 29 genes)	20.3%	6.96 (95% CI 2.57–18.91) *p* < 0.001
Scholer et al. [80]	21	Stage I–III CRC	Weeks 1–4 post-op	ddPCR (NA)	28.5%	37.7 (95% CI 4.2–335.5) *p* < 0.001

CRC: colorectal cancer, CC: colon cancer, NA: non-available, ctDNA: circulating tumor DNA, RFS: recurrence free survival, PCR: polymerase chain reaction, ddPCR: droplet digital PCR, HR: hazard ratio, CI: confidence interval, NGS: next-generation sequencing, Post-op: post-surgery, Pre-op: pre-surgery.

**Table 2:** ctDNA as prognostic marker in locally advanced rectal cancer.

Study	Sample Size	Study Population	Timepoint of ctDNA Collection	ctDNA Detection Assay	Baseline ctDNA Detection Rate	Pre-op ctDNA Detection Rate	Post-op ctDNA Detection Rate	Main Results for ctDNA+ vs. ctDNA−
Tie et al. [84]	159	LARC	Pre-treatment (CRT), weeks 4–6 post-CRT, and weeks 4–10 post-op	Safe-SeqS (1 variant; 15 genes)	77%	8.3%	12%	Post-CRT RFS: HR 6.6 (95% CI 2.6–17) *p* < 0.001 Post-op RFS: HR 13 (95% CI 5.5–31) *p* < 0.001
Khakoo et al. [85]	47	Localized rectal cancer	Pre-treatment (CRT), mid-CRT, post-CRT, and weeks 4–12 post-op	ddPCR (up to 3 variants; 6 genes)	74%	21%	13%	Post-CRT MFS: 7.1 (95% CI 2.4–21.5) *p* < 0.001 Post-op DFS: 39.9 (95% CI 4.0–399.5) *p* = 0.002
Zhou et al. [86]	104	LARC	Pre-treatment (CRT), 1 week from the start of treatment, post-CRT, and 4 weeks post-op	HiSeq 3000 Sequencing System (Illumina^TM^). Panel of 1021 genes	75%	10.5%	6.7%	Post-CRT MFS:19.82 (95% CI 2.029–193.7) *p* < 0.001 Post-op MFS: 25.30 (95% CI, 1.475–434) *p* < 0.001
Murahashi et al. [87]	85	LARC	Pre-treatment (CRT), post-CRT, and 12 weeks post-op	Oncomine CRC (14 genes)	57.6%	22.3%	NA	Post-op RFS: 17.1 (95% CI, 1.0–282) *p* < 0.001
Pazdirek et al. [88]	36	LARC	Pre-treatment (CRT), 1 week from the start of treatment	Denaturing capillary electrophoresis (DCE) and High sensitivity Beaming assay	21.2%	NA	NA	Prior CRT: reduction DFS by 1.47 years (*p* = 0.015) and OS by 1.41 years (*p* = 0.010)
Vidal et al. [89]	62	LARC	Pre-treatment (TNT) and post-CRT (48 h pre-op)	LUNAR-1	83%	15%	NA	Post-CRT RFS: HR 4.029 (95% CI, 1.004–16.16) *p* = 0.033 Post-CRT OS: HR 23(95% CI, 2.4–212) *p* < 0.0001

LARC: locally advanced rectal cancer, CRC: colorectal cancer, CRT: chemo-radiotherapy, TNT: total neoadjuvant treatment, MFS: metastasis free-survival, ctDNA: circulating tumor DNA, RFS: recurrence free survival, ddPCR: droplet digital PCR, HR: hazard ratio, CI: confidence interval, Post-op: post-surgery, Pre-op: pre-surgery.

**Table 3:** ctDNA−guided ongoing clinical trials in early-stage colorectal cancer.

Trial Name	Sample Size	Study Design	Study Population	Timepoint of ctDNA Collection	ctDNA Detection Assay	Design	ctDNA Treatment Intervention
DYNAMIC (ACTRN12615000381583) Australia/NZ	450	NA	Stage II CRC	Week 4 post-op	Safe-SeqS (1 variant; 15 genes)	Randomization 1:1 SOC- vs. ctDNA− guided treatment	ctDNA+: 5FU-based regimen ± oxaliplatin for 3–6 months; ctDNA− no chemotherapy
DYNAMIC III (ACTRN12617001566325) Australia/NZ	1000	Phase II/III	Stage III CC	Weeks 5–6 post-op	Safe-SeqS (1 variant; 15 genes)	Randomization 1:1 SOC- vs. ctDNA− guided treatment	ctDNA+: escalated chemotherapy regimen from pre-planned treatment (increase duration or number of agents); ctDNA−: de-escalated chemotherapy regimen from pre-planned treatment (reduction in duration or number of agents)
DYNAMIC RECTAL (ACTRN12617001560381) Australia/NZ	408	NA	LARC	Week 4 post-op	Safe-SeqS (1 variant; 15 genes)	Randomization 1:1 SOC vs. ctDNA guided	ctDNA+: adjuvant chemotherapy ctDNA− and ypN0: surveillance ctDNA− and ypN+ surveillance or adjuvant chemotherapy at clinician’s choice
CIRCULATE (NCT04089631) Germany	4812	Phase III	Stage II CRC	Week 5 post-op	NGS (NA)	ctDNA+ randomization 2:1 chemotherapy vs. follow-up	Capecitabine × 6 months vs. follow-up
CIRCULATE-PRODIGE 70 (NCT04120701) France	198	Phase III	Stage II (pT3-pT4aN0) CRC	Week 2 post-op	ddPCR (2 methylated markers)	ctDNA+ randomization 2:1 chemotherapy vs. follow-up	mFOLFOX (× 6 months) vs. follow-up
COBRA (NCT04068103) US	1408	Phase II/III	Stage II (low risk CC)	NA	Guardant LUNAR-1 (NA)	Randomization 1:1 surveillance- vs. ctDNA− guided treatment	ctDNA+: CAPOX or FOLFOX vs. ctDNA−: no chemotherapy
TRACC (NCT04050345) UK	1000	Observational study	Stage II/III CRC	Weeks 4–8 post-op	Customized NGS panel (NA)	Randomization 1:1 SOC- vs. ctDNA− guided treatment	ctDNA+: SOC ctDNA−: de-escalate treatment (from 3 months CAPOX to 3 months Capecitabine and from 6 months capecitabine to no chemotherapy) but re-escalate if ctDNA becomes positive at 3 months
(UMIN000039205) Japan	1240	NA	High-risk stage II/low-risk III CRC	Week 4 post-op	Signatera-PCR NGS assay (16 specific somatic variants)	ctDNA− randomization SOC vs. no treatment	ctDNA− Randomization SOC vs. no treatment
MEDOCC-CrEATE (NL6281/NTR6455) Netherlands	1320	NA	Stage II (low risk CC)	Weeks 1–3 post-op	PGDx elio (panel of more than 30 genes)	Randomization 1:1 SOC- vs. ctDNA− guided treatment	ctDNA+: 6 months CAPOX or FOLFOX; ctDNA−: no chemotherapy
PEGASUS (NCT04259944) Italy and Spain	140	Phase II	High-risk stage II/III CC	Weeks 2–4 post-op	Guardant LUNAR-1 (NA)	ctDNA− guided treatment	ctDNA+: CAPOX × 3 months → 2nd ctDNA+ switch FOLFIRI; secind ctDNA− capecitabine × 3 month ctDNA−: capecitabine × 6 months. 2nd ctDNA+ → CAPOX × 6 months
ACT-3 trial (NCT04259944)	500	NA	Stage III CRC	Weeks 3–6 post-op and 3–6 months after adjuvant treatment	Guardant LUNAR-1 (NA)	ctDNA− guided treatment	ctDNA+ after adjuvant treatment: randomized to follow-up or molecular target-directed therapy ctDNA−: follow-up
ALTAIR trial (UMIN000039205)Japan	240	NA	Stage II/III CRC or stage IV with resectable metastases	1 month after surgery and 3 months after standard adjuvant treatment	Signatera-PCR NGS assay	Signatera-PCR NGS assay	ctDNA+ after adjuvant treatment: randomized to follow-up or second-line trifluridine/Tipiracil ctDNA−: follow-up

LARC: locally advanced rectal cancer, CRC: colorectal cancer, CC: colon cancer, NA: non-available, Post-op: post-surgery, ctDNA: circulating tumor DNA, RFS: recurrence free survival, PCR: polymerase chain reaction, ddPCR: Droplet digital PCR, HR: hazard ratio, CI: confidence interval, NGS: next-generation sequencing, NZ: New Zealand, US: United States, UK: United Kingdom.
